# Supplementary material for: Targeting ADAR stability: a novel approach to combat glioblastoma
Source: Front Oncol. 2026 Aug 6;16:1827793. doi: 10.3389/fonc.2026.1827793 (PMC13489870; doi:10.3389/fonc.2026.1827793)
Supplement: Supplementary file 1 [file SupplementaryFile1.zip › Supplementary file 1/One_carbon_pool_by_folate.pdf]

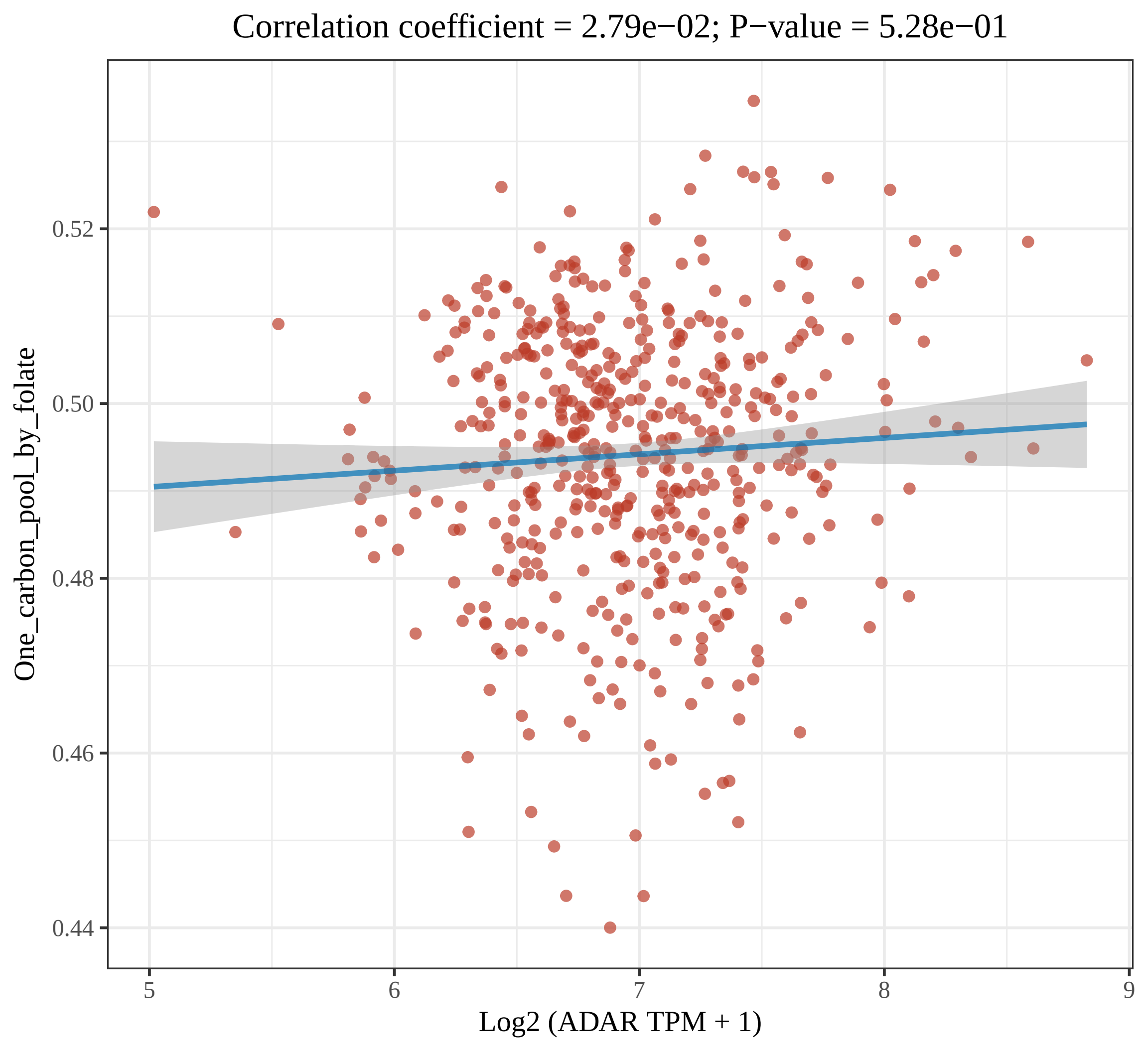
Correlation coefficient = 2.79e−02; P−value = 5.28e−01
One_carbon_pool_by_folate
0.44
0.46
0.48
0.50
0.52
5
6
7
8
9
Log2 (ADAR TPM + 1)
